# A structural overview of the zinc transporters in the cation diffusion facilitator family

**DOI:** 10.1107/S2059798319003814

**Published:** 2019-04-05

**Authors:** Camila A. Cotrim, Russell J. Jarrott, Jennifer L. Martin, David Drew

**Affiliations:** a Griffith Institute for Drug Discovery, Griffith University, Nathan, QLD 4111, Australia; b Department of Biochemistry and Biophysics, Stockholm University, SE-106 91 Stockholm, Sweden

**Keywords:** cation diffusion facilitator, membrane proteins, zinc transporter

## Abstract

The cation diffusion facilitators (CDFs) are a family of membrane-bound proteins that maintain cellular homeostasis of essential metal ions. In humans, the zinc-transporter CDF family members (ZnTs) play important roles in zinc homeostasis. They do this by facilitating zinc efflux from the cytoplasm to the extracellular space across the plasma membrane or into intracellular organelles. Several ZnTs have been implicated in human health owing to their association with type 2 diabetes and neurodegenerative diseases. Although the structure determination of CDF family members is not trivial, recent advances in membrane-protein structural biology have resulted in two structures of bacterial YiiPs and several structures of their soluble C-terminal domains. These data reveal new insights into the molecular mechanism of ZnT proteins, suggesting a unique rocking-bundle mechanism that provides alternating access to the metal-binding site.

## Abstract 2

A summary is provided of the last five years of structural studies of bacterial YiiP, providing an overview of how zinc-transporter membrane proteins operate at the molecular level.

## 1. Introduction   

Zinc (Zn^2+^) is a key metal for important cellular processes including immune function, redox signalling, growth and cell death (Vallee & Falchuk, 1993 ▸). At least 10% of all mammalian proteins are predicted to bind Zn^2+^ (Andreini *et al.*, 2006 ▸; Maret, 2008 ▸); when bound to a protein, Zn^2+^ can have structural or catalytic roles (Andreini & Bertini, 2012 ▸). A 2013 study estimated that more than 17% of the global population is at risk of inadequate zinc intake (Wessells & Brown, 2012 ▸). Zinc deficiency causes diarrhoea, chronic inflammation and growth retardation (Prasad, 2013 ▸), and has been implicated in medical conditions such as diabetes and Alzheimer’s disease (Lovell *et al.*, 2005 ▸; Sladek *et al.*, 2007 ▸). In plants, Zn^2+^ deficiency has been associated with growth disorders, plant mortality and inhibition of flowering (Pandey *et al.*, 2006 ▸; Wissuwa *et al.*, 2006 ▸). Despite its importance for health and growth, elevated levels of intracellular Zn^2+^ are toxic, and therefore its concentration in cells must be tightly regulated (Huang & Tepaamorndech, 2013 ▸).

To date, four major families of proteins associated with zinc homeostasis have been identified: (i) metallothioneins, (ii) Zn^2+^-transporting P-type ATPases, (iii) ZRT/IRT-like proteins (ZIPs) and (iv) cation diffusion facilitators (CDFs) (Kolaj-Robin *et al.*, 2015 ▸; Blindauer, 2015 ▸; Kimura & Kambe, 2016 ▸). Metallothioneins are soluble proteins that are found in eukaryotes, plants, fungi and bacteria (Blindauer, 2015 ▸). These proteins contribute to cellular zinc homeostasis by chelating free Zn^2+^ and lowering its intracellular concentration (Kimura & Kambe, 2016 ▸). Zn^2+^-transporting P-type ATPases are membrane proteins that are present in plants and bacteria, including several pathogens (Sitsel *et al.*, 2016 ▸). Many P-type ATPases are involved in Zn^2+^ efflux, transporting the metal from the cytosol into the intracellular compartments (Blindauer, 2015 ▸). ZIP and CDF are ubiquitous families of proteins that are found across all major phyla (Kolaj-Robin *et al.*, 2015 ▸; Jeong & Eide, 2013 ▸). ZIP proteins mediate the uptake of Zn^2+^ into the cytosol either from the extracellular space or from organelles (Bafaro *et al.*, 2017 ▸).

CDF proteins are involved in the homeostasis of transition-metal ions and tolerance to trace elements (Nies & Silver, 1995 ▸). The family was initially divided into three broad groups (Montanini *et al.*, 2007 ▸). Group 1 (zinc-CDFs) consists of Zn^2+^ and Co^2+^ transporters such as the ZitB-like, ZnT1-like and Zrc1-like clusters. Group 2 (iron/zinc-CDFs) includes the well known YiiP (FieF) from *Escherichia coli*. Members of this group have been reported to transport Fe^2+^ or Zn^2+^, and also Co^2+^, Cd^2+^ and Ni^2+^. Group 3 (manganese-CDFs) includes some metal-transporter proteins (MTP) from plants (Montanini *et al.*, 2007 ▸). Recently, a phylogenomic study suggested a new diversification for group 2, separating this group into 18 independent clades according to the metal specificity, with Zn^2+^/Cd^2+^, Co^2+^/Ni^2+^, Fe^2+^ and Zn^2+^/Cd^2+^/Fe^2+^/Mn^2+^ groups (Cubillas *et al.*, 2013 ▸).

Members of the CDF family contain an N-terminal domain, a transmembrane domain formed by six helices and a long C-terminal domain (CTD) (Paulsen & Saier, 1997 ▸; Kolaj-Robin *et al.*, 2015 ▸). Numerous putative CTD-lacking CDF proteins, the function of which is still unclear, have been identified in marine bacteria, soil bacteria and pathogens (Kolaj-Robin *et al.*, 2015 ▸). Several eukaryotic proteins also contain an additional histidine-rich loop between TM4 and TM5 (Paulsen & Saier, 1997 ▸) that is believed to be involved in zinc coordination during zinc transport (Fukue *et al.*, 2018 ▸). This review focuses on the CDF members for which structures have been solved and summarizes our knowledge of their structure–function relationship.

## 2. The CDF transporters in humans   

Mammalian CDFs are named zinc transporters (ZnTs) or solute carrier family 30 (SLC30A), and a total of ten ZnT proteins (ZnT1–ZnT10) have been identified (Huang & Tepaamorndech, 2013 ▸). ZnT transporters, together with ZIP proteins, control the mobilization of zinc across biological membranes and maintain cytosolic levels of free zinc in the picomolar concentration range (Maret, 2013 ▸). Briefly, ZIP proteins facilitate the mobilization of zinc into the cytosol from the extracellular space or intracellular compartments, whereas ZnT proteins facilitate zinc efflux out of the cytosol into the extracellular space or intracellular compartments (Huang & Tepaamorndech, 2013 ▸; Kimura & Kambe, 2016 ▸).

ZnTs can be divided into four subfamilies based on their sequence similarity. ZnT5 and ZnT7 are in subfamily I, ZnT2, ZnT3, ZnT4 and ZnT8 are grouped in subfamily II, ZnT1 and ZnT10 are in subfamily III, and subfamily IV contains ZnT6 and the contentious ZnT9 (Huang & Tepaamorndech, 2013 ▸). Previous studies have suggested that ZnT9 is not a zinc transporter at all, and is only involved in Wnt signalling (Chen *et al.*, 2007 ▸). A more recent study has shown that ZnT9 has dual activity, acting as a zinc transporter and participating in Wnt signalling (Perez *et al.*, 2017 ▸).

**Figure 1:**
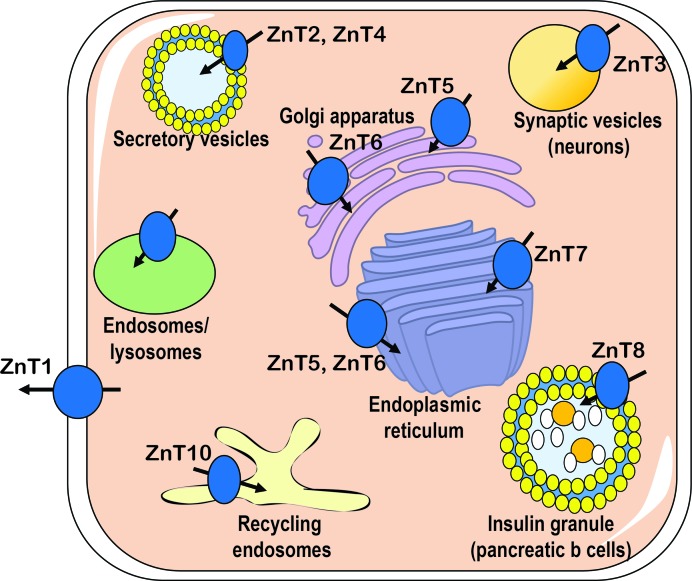
Subcellular localization of mammalian ZnT transporters. Cytosolic zinc is mobilized into or out of a compartment through the action of ZnT, as indicated by the arrows. This figure was adapted from Chabosseau & Rutter (2016 ▸).

ZnTs are distributed differently in different tissues. ZnT1 is the only exporter of the ZnT family that is primarily localized to the plasma membrane, and therefore plays a major role in transporting zinc from the cytoplasm, across the plasma membrane and into the extracellular space (Qin *et al.*, 2009 ▸). However, it should be noted that several studies have reported that isoforms of ZnT2, ZnT5, ZnT8 and ZnT10 are localized to the plasma membrane under certain physiological conditions (Jackson *et al.*, 2007 ▸; Lopez & Kelleher, 2009 ▸; Bosomworth *et al.*, 2012 ▸; Huang *et al.*, 2017 ▸; Carvalho *et al.*, 2017 ▸). The subcellular localization of ZnT transporters has been reviewed previously (Liuzzi & Cousins, 2004 ▸; Kambe *et al.*, 2015 ▸) and is summarized in Fig. 1 ▸.

Several mammalian ZnT transporters have been described as antiporters in that they transport metal by a proton-motive force, acting as *M* ^2+^/H^+^ antiporters (Ohana *et al.*, 2009 ▸; Shusterman *et al.*, 2014 ▸; Chao & Fu, 2004*a* ▸; Guffanti *et al.*, 2002 ▸). To date, no three-dimensional structure is available of any eukaryotic ZnT transporter or CDF member, except for a predicted structure of ZnT8 based on modelling from the crystal structure of the *E. coli* homolog YiiP (Weijers, 2010 ▸; Lu & Fu, 2007 ▸). Moreover, most ZnT proteins are predicted to form functional homodimers (Kambe, 2012 ▸), except for ZnT5 and ZnT6, which have been reported to be heterodimers (Ohana *et al.*, 2009 ▸; Fukunaka *et al.*, 2009 ▸).

Over the last decades, much attention has been paid to ZnTs owing to their association with severe diseases. Zinc has been implicated in neurodegenerative diseases, including Parkinson’s disease and Alzheimer’s disease (AD). Numerous studies have linked altered expression levels of ZnT1, ZnT3 and ZnT6 and an increased intracellular concentration of Zn^2+^ to the initiation of amyloid-β peptide (Aβ) deposition and senile plaque formation in the AD brain (Lyubartseva *et al.*, 2010 ▸; Lovell *et al.*, 2005 ▸; Lee *et al.*, 2002 ▸; Zhang *et al.*, 2008 ▸). In addition, dysregulation of ZnT10 levels in the frontal cortex in the AD brain has also been suggested to contribute to disease progression (Bosomworth *et al.*, 2013 ▸). Relevant studies have also focused on ZnT2 after a missense mutation on the *SLC30A2* gene, causing a histidine-to-arginine (H54R) change, was associated with transient neonatal zinc deficiency (TNZD) (Chowanadisai *et al.*, 2006 ▸). Itsumura and coworkers reported three novel ZnT2 mutations associated with TNZD that inhibited the transport of zinc by ZnT2 (Itsumura *et al.*, 2016 ▸). Furthermore, altered levels of ZnT2 and other zinc transporters have been reported in breast cancer cells, linking this condition to dysregulated zinc homeostasis (Chandler *et al.*, 2016 ▸). The implications of zinc deficiency and the effect of zinc transporters on different types of cancers have been reviewed by Pan *et al.* (2017 ▸).

ZnT8 has also been the target of many studies since genome-wide association analyses linked it to an altered risk of type 2 diabetes (T2D). Sladek and coworkers reported that a common single-nucleotide polymorphism on the *SLC30A8* gene, which leads to the replacement of an arginine by a tryptophan (R325W), confers a 15% increased risk of developing T2D (Sladek *et al.*, 2007 ▸). Conversely, a 2014 study identified 12 rare loss-of-function ZnT8 variants that have protective effects and decrease the risk of T2D by 65% (Flannick *et al.*, 2014 ▸).

Despite the challenges involved in the membrane-protein structural biology field, the three-dimensional structure of the bacterial protein YiiP, a CDF member, has been determined by X-ray crystallography and by cryo-electron microscopy, providing new insights into the mechanism of action of these transporters.

## 3. The structure of YiiP: a bacterial member of the CDF family   

YiiP was initially named as a ferrous iron efflux (FieF) protein owing to its positive correlation with the intracellular iron concentration (Grass *et al.*, 2005 ▸). However, its primary role is to transport Zn^2+^ and Cd^2+^ across membranes (Grass *et al.*, 2005 ▸; Wei & Fu, 2005 ▸; Chao & Fu, 2004*b* ▸), although iron efflux transport has been reported using purified protein reconstituted in proteoliposomes and YiiP can promote iron detoxification only (Grass *et al.*, 2005 ▸).

**Figure 2:**
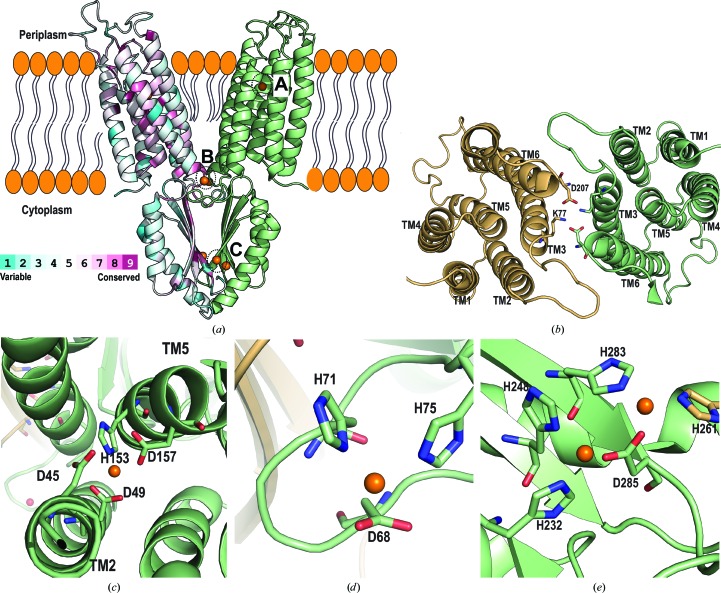
Crystal structure of EcYiiP. (*a*) YiiP homodimer from *E. coli* (PDB entry 3h90) crystallized in a detergent micelle and represented in a lipid bilayer plane. Chain *A* is coloured according to sequence conservation generated using the *ConSurf* server, with turquoise through maroon indicating variable through conserved (Ashkenazy *et al.*, 2016 ▸), on the basis of an alignment of YiiP and human zinc transporters (ZnTs). Chain *B* is coloured light green. Zn^2+^ ions are shown as orange spheres at the sites labelled A, B and C. (*b*) View of the structure from the periplasmic side, showing the bundle of four helices and the bundle of two helices and the salt bridges formed by (Lys77–Asp207)_2_. The CTDs have been removed for clarity. (*c*, *d*, *e*) Binding sites A, B and C, respectively, with bound Zn^2+^ (orange spheres) and coordination residues (shown in stick representation and labelled).

YiiP belongs to the CDF family (Paulsen & Saier, 1997 ▸). Because of its ability to transport Zn^2+^ in a proton-dependent manner, it is one of two antiporters (along with ZitB) that control zinc homeostasis in *E. coli* (Grass *et al.*, 2005 ▸). YiiP from *E. coli* has been shown to be dimeric both in detergent micelles and in the lipid bilayer membrane (Wei *et al.*, 2004 ▸). The X-ray crystallographic structure of YiiP from *E. coli* (EcYiiP) in a detergent micelle was first solved to 3.8 Å resolution (Lu & Fu, 2007 ▸) and was later improved to 2.9 Å resolution (Lu *et al.*, 2009 ▸). The crystal structure of EcYiiP reveals a Y-shaped homodimer with each of the two arms formed by the transmembrane domains (TMDs) of the two protomers. These TMD arms are splayed apart and form no intermolecular interactions (Fig. 2 ▸ *a*). Each monomer consists of six transmembrane helices and a cytosolic CTD. The CTDs juxtapose with each other in parallel and contribute the dimerization contacts. The six transmembrane helices can be grouped into two independent subdomains: a bundle of four involving TM1, TM2, TM4 and TM5, and a bundle of two involving TM3 and TM6. The latter two TMD helices cross over in an antiparallel manner and this is stabilized by two salt bridges formed between Lys77 of TM3 and Asp207 of TM6 (Lu *et al.*, 2009 ▸; Lu & Fu, 2007 ▸; Fig. 2 ▸ *b*).

These first crystal structures identified three highly conserved zinc-binding sites (termed A, B and C). Binding site A is located between TM2 and TM5, and Zn^2+^ is tetracoordinated by Asp45 and Asp49 from TM2 and His153 and Asp157 from TM5 (Lu & Fu, 2007 ▸; Lu *et al.*, 2009 ▸; Fig. 2 ▸ *c*). These residues are conserved and are essential for Zn^2+^ and Cd^2+^ transport (Wei & Fu, 2006 ▸). Binding site B is close to one of the dimer interfaces and is located in the loop between TM2 and TM3 on the cytoplasmic membrane. Zn^2+^ is coordinated by Asp68, His71 and His75 (Fig. 2 ▸ *d*). Binding site C has the highest metal affinity (Lu & Fu, 2007 ▸; Lu *et al.*, 2009 ▸; Coudray *et al.*, 2013 ▸) and is located at the CTD–CTD interface. It harbours four Zn^2+^ ions in total, two from each monomer, and these contribute to dimer stabilization. The Zn^2+^ ions are bridged by the conserved Asp285 and are coordinated by a series of histidines (His232, His248 and His283 from the same protomer, and His261 from the neighbouring protomer; Fig. 2 ▸ *e*).

Studies on YiiP from another bacterium provided additional information about the three-dimensional structure of this protein family. The cryoelectron microscopy (cryo-EM) structure of YiiP from *Shewanella oneidensis* (SoYiiP) was solved within a lipid environment at 13 Å resolution, with the zinc-binding sites thought to be occupied by H^+^ (Coudray *et al.*, 2013 ▸). Similar to the YiiP structure from *E. coli*, SoYiiP forms a dimer, with the cytoplasmic domain of EcYiiP fitting easily into the cryo-EM density of SoYiiP. Interestingly, the data show that the transmembrane domains of SoYiiP are closer together than in the Zn^2+^-bound YiiP structure; major conformational changes are required to fit the EcYiiP transmembrane domains from the crystal structure into the cryo-EM data for SoYiiP. Comparison of the cryo-EM model with the X-ray structure suggested that the Zn^2+^-transport sites are accessible from the periplasmic side in the EcYiiP crystal structure, which is consistent with the outward-facing state described for many secondary transporters (Jardetzky, 1966 ▸), whereas the cryo-EM model of SoYiiP is consistent with an inward-facing state (Coudray *et al.*, 2013 ▸).

More recently, another cryo-EM structure of SoYiiP was solved at 4.1 Å resolution, providing new details about the structure of YiiP (Lopez-Redondo *et al.*, 2018 ▸). Although the overall architecture of the higher resolution SoYiiP structure is very similar to the previous lower resolution structure of SoYiiP, the higher resolution data revealed differences in the conformations of the TM helices. The impact of these differences on the proposed mechanism of action of YiiP will be discussed in Section 5.

## 4. Metal selectivity among the CDF proteins   

A better understanding of the metal selectivity and specificity of the CDF transporters is essential to define the molecular mechanism of these proteins. Although studies have elucidated the three-dimensional structures of some CDF members, little is known concerning the metal selectivity of most CDF transporters. The metal selectivity of YiiP, however, has been well established. Recent studies have reported that purified YiiP selects Zn^2+^ and Cd^2+^ against Fe^2+^ and other divalent metal ions (Hoch *et al.*, 2012 ▸).

As mentioned above, numerous eukaryotic CDF proteins, including some mammalian zinc transporters and metal-tolerance proteins (MTPs) in plants, contain a His-rich region between TM4 and TM5 (Paulsen & Saier, 1997 ▸), which was first thought to be involved in zinc binding (Williams *et al.*, 2000 ▸). This finding was confirmed by studies on the MTP1s from *Arabidopsis thaliana* (AtMTP1) and barley (HvMTP1) (Kawachi *et al.*, 2008 ▸; Podar *et al.*, 2012 ▸). HvMTP1 is a Zn^2+^- and Co^2+^-specific transporter, while AtMTP1 only transports Zn^2+^. Working with AtMTP1, Kawachi and coworkers showed that a mutant AtMTP1 lacking 32 residues of the His-loop had increased activity, suggesting that the loop functions as a Zn^2+^ ion sensor (Kawachi *et al.*, 2008 ▸). Additional studies comparing the His-rich loops of AtMTP1 and HvMTP1 identified five residues (VTVTT) within this region that limit AtMTP1 to transport Zn^2+^ only, suggesting that the His-rich region is involved in metal selectivity as well as zinc sensing and binding (Podar *et al.*, 2012 ▸).

Further studies with EcYiiP and human zinc transporters 5 and 8 (ZnT5 and ZnT8) have suggested that a tetrahedral metal-transport motif is critical for metal selectivity (Hoch *et al.*, 2012 ▸). The crystal structure of YiiP revealed a tetrahedral transport site (binding site A; Figs. 2 ▸ *c* and 3 ▸) with the composition Asp45-Asp49–His153-Asp157 (DD-HD), whereas the human orthologs, which are very specific for Zn^2+^, have an HD-HD binding site. Hoch and coworkers reported that the Zn^2+^ and Cd^2+^ specificity of YiiP can be changed to Zn^2+^ only by the mutation of the first aspartate of the **D**D-HD motif to histidine (**H**D-HD). Conversely, the mutation of human ZnT transporters to a more ‘YiiP-like’ protein abolished selectivity against Cd^2+^ with no effect on Zn^2+^ transport (Hoch *et al.*, 2012 ▸). The finding that histidine plays a role in selectivity for Zn^2+^ over Cd^2+^ in ZnTs can be attributed to the coordination chemistry of these metals. Zinc prefers tetrahedral binding and the histidine residue of binding site A may be sufficient to stabilize Zn^2+^. Cadmium, on the other hand, prefers tetrahedral and octahedral coordination geometries (Barber-Zucker *et al.*, 2017 ▸). Moreover, analysis of metal-coordination geometry suggests that Cd^2+^ has a higher probability of binding to glutamic acid and aspartic acid than to histidine, whereas Zn^2+^ tends to coordinate with histidine and cysteine (Barber-Zucker *et al.*, 2017 ▸).

**Figure 3:**
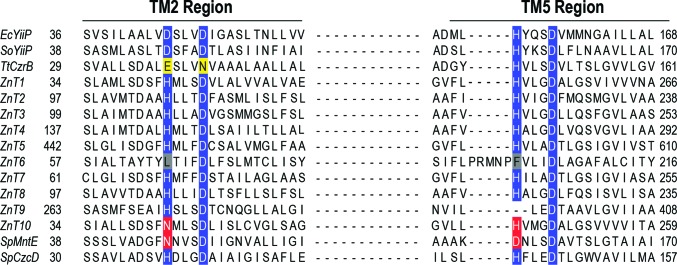
Sequence alignment of the tetrahedral motifs. The zinc-binding motifs of human ZnTs and bacterial CDF members are shown. The alignment was prepared using *MUSCLE* (Edgar, 2004 ▸) and *Jalview* (Waterhouse *et al.*, 2009 ▸). The tetrahedral motif is mostly highlighted in blue. Red highlighting indicates the different residues in the two CDF proteins associated with Mn^2+^ transport. Yellow and grey highlighting indicates residues that differ from the standard (D or H) residues in the tetrahedral motif. TM, transmembrane. Accession numbers are as follows: *Escherichia coli* YiiP (EcYiiP), P69380; *Shewanella oneidensis* YiiP (SoYiiP), Q8E919; *Thermus thermophilus* CzrB (TtCzrB), Q8VLX7; ZnT1, Q9Y6M5; ZnT2, Q9BRI3-2; ZnT3, Q99726; ZnT4, O14863; ZnT5, Q8TAD4; ZnT6, Q6NXT4; ZnT7, Q8NEW0; ZnT8, Q8IWU4; ZnT9, Q6PML9; ZnT10, Q6XR72; *Streptococcus pneumoniae* MntE (SpMntE), Q8DP19; *S. pneumoniae* CzcD (SpCzcD), A0A0B7LW62.

Martin and Giedroc investigated the role of the tetrahedral motif of binding site A on the metal selectivity of other CDF members. Their study focused on the CzcD and MntE proteins from *Streptococcus pneumoniae*, which are Zn^2+^ and Mn^2+^ transporters, respectively. Their results suggested that metal selectivity is mainly dictated by two residues within the tetrahedral motif. According to their analysis, asparagine and aspartic acid (**N**D-**D**D) give manganese specificity, whereas a histidine pair (**H**D-**H**D) gives zinc specificity (Martin & Giedroc, 2016 ▸). Similar results have been observed for the human ZnT10 transporter, which was shown to be associated with manganese rather than zinc efflux. The ZnT10 protein sequence has an **N**D-**H**D motif (Fig. 3 ▸), suggesting that this motif, together with two other residues from TM2 and TM5, might control metal specificity. This notion was further supported by the His–Asn reversion mutant of ZnT1, which has no zinc-transport activity but does transport manganese (Nishito *et al.*, 2016 ▸). Conversely, a parallel study on human ZnT10 metal specificity reported that the asparagine in the **N**D-HD motif is not essential for Mn^2+^ efflux activity. Interestingly, the authors of this study reported that within the tetrahedral motif, mutation of only the last aspartate Asp248 (ND-H**D**) was necessary to abolish ZnT10 activity, suggesting that asparagine does not play a role in Mn^2+^ specificity. In addition, they also demonstrated that residues outside the tetrahedral motif (Gly25 and Asn127) are required for manganese efflux, suggesting that the orientation of these residues might contribute to the octahedral coordination of Mn^2+^ and disfavour zinc coordination (Zogzas *et al.*, 2016 ▸). Whilst the conflicting conclusions from these two studies can potentially be explained by differences in the cell lines used for the functional assays and the lack of metal-measurement data (Zogzas *et al.*, 2016 ▸), this discrepancy once again highlights the need for additional CDF structures to more fully investigate the mechanism of action and the roles that specific residues play in metal binding and transport.

A recent *in silico* study reported the correlation between the tetrahedral motif across the entire CDF family and their differential metal selectivity and their phylogenetic classification (Barber-Zucker *et al.*, 2017 ▸). By analysing the 18 clades previously described (Cubillas *et al.*, 2013 ▸), Barber-Zucker and coworkers identified clades with distinctive signatures, although no reasonable correlations could be determined between the clades, the tetrahedral motif and the metal selectivity. Some eukaryotic clades containing the His pair (HD-HD) predominantly showed zinc-transport activity. However, a distinct clade that conserves the HD-HD motif, and includes a mixture of bacterial, archaeal and eukaryotic CDF proteins, has been linked to iron transport, suggesting that other residues in addition to this motif may dictate metal selectivity (Barber-Zucker *et al.*, 2017 ▸).

## 5. The proposed mechanism of action of YiiP and implications for dimerization   

Dimerization is a common feature of CDF transporters and is therefore thought to be involved in functionality (Eide, 2006 ▸). In 2009, Lu and coworkers proposed two factors that mediate dimer stabilization: (i) the interface between the cytoplasmic CTDs, where two zinc ions per monomer are required to keep the domains together, and (ii) a salt bridge at the cytoplasmic membrane surface, called the charge interlock, consisting of four residues (Lys77–Asp207)_2_ in the TM3 and TM6 pairs (Fig. 2 ▸ *b*; Lu *et al.*, 2009 ▸). These two features, together with the YiiP crystal structure, provided the basis for the first proposed mechanism of action: a scissoring model that involves the CTDs.

**Figure 4:**
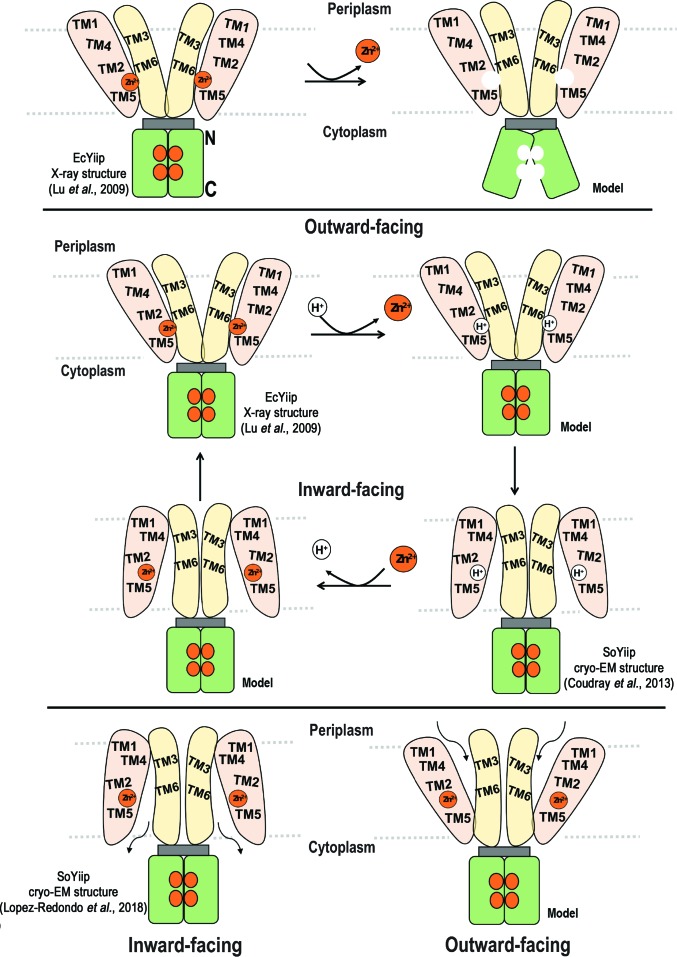
Structural/mechanistic models. (*a*) Schematic showing the scissoring mechanism proposed for zinc transfer by YiiP. The bundle of four helices (TM1, TM2, TM4 and TM5) is shown in light orange, while the bundle of two helices (TM3 and TM6) is shown in light yellow. The charge interlock (shown in grey) supports the scissoring mechanism of the CTD (shown in green) which is triggered by the release of Zn^2+^ ions. The N- and C-­termini of the CTD are indicated in the left panel. The proposed mechanism is based on the crystal structure of EcYiiP and FRET measurements. This figure is adapted from Lu *et al.* (2009 ▸). (*b)* Alternating-access mechanism proposed by Coudray *et al.* (2013 ▸). The mechanism, based on the crystal structure of EcYiiP and the cryo-EM structure of SoYiiP, involves a proton- and zinc-bound helical bundle and inward-facing and outward-facing conformations. Major conformational changes in both helical bundles (light orange and yellow) are proposed on moving between the four states. In this model, the CTD (green) remains static and the dimer interface is mediated by four CTD-bound Zn^2+^ ions. (*c*) Model of the inward-facing and outward-facing conformations of the zinc-bound state of YiiP. Transport is driven by rearrangements of the four-helix bundle (light orange) relative to a static TM3–TM6 scaffold (light yellow). For these models, the inward-facing conformation corresponds to the cryo-EM structure of SoYiiP, whereas the outward-facing conformation corresponds to the X-ray structure of EcYiiP after adjusting the TMs to adopt a compact configuration.

According to the postulated mechanism, the TMD–TMD hydrophobic interactions and the charge interlock provide a favourable electrostatic environment for Zn^2+^ to bind to the CTDs (site C), bringing the two protomers together. Upon Zn^2+^ release, the CTDs are driven apart by charge repulsion (Lu *et al.*, 2009 ▸). The scissoring mechanism is supported by the charge interlock, which serves as a pivot point for movements between the CTDs and alterations in TM3–TM6 (Fig. 4 ▸ *a*). Consequently, TM5 is also re­oriented owing to its packing contacts with TM3–TM6, whilst TM2 remains static (Lu *et al.*, 2009 ▸). Structural comparisons of the monomeric EcYiiP CTD with the soluble CTD of CzrB, a metal transporter from *Thermus thermophilus* (Spada *et al.*, 2002 ▸), showed minimal structural changes in the presence or absence of zinc (Cherezov *et al.*, 2008 ▸; Lu *et al.*, 2009 ▸). This finding suggested that structural modifications of the dimeric domain might occur ‘en bloc’ rather than as small or local conformational changes.

**Figure 5:**
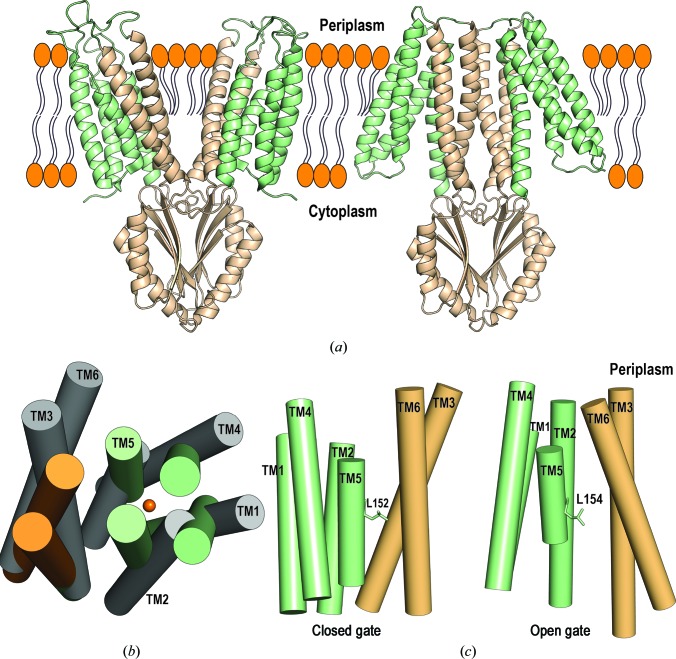
Comparison of EcYiiP and SoYiiP structures. (*a*) Structural comparison of the crystal structure of *E. coli* YiiP (left; PDB entry 3h90; Lu *et al.*, 2009 ▸) and the cryo-EM structure of *S. oneidensis* YiiP (right; PDB entry 3j1z; Coudray *et al.*, 2013 ▸), showing that the transmembrane domains in the cryo-EM structure adopt a more compact conformation. TM3 and TM6 and the CTD are coloured wheat. (*b*) Overlay of the transmembrane helices of EcYiiP (grey) and SoYiiP (light green and wheat), showing that conformational changes may occur between the bundle of four and the bundle of two TM helices. View from the periplasm. (*c*) Representation of the Leu152 gate. In the SoYiiP structure (open gate) the orientation of Leu154 (analogous residue) exposes the binding site to the aqueous bulk, whereas TM5 undergoes conformational changes upon zinc binding, leading Leu152 to close the gate.

In 2013, six years after the crystal structure of EcYiiP had been reported, the cryo-EM structure of SoYiiP (49% sequence identity) was described. The SoYiiP structure, described as representing the protein with binding sites occupied by H^+^, revealed a very different orientation of the TMDs within the membrane compared with the EcYiiP structure. Specifically, the TMDs of the two protomers were more closely associated with each other (Fig. 5 ▸ *a*), suggesting that a conformational change in the TMDs may be relevant to the mechanism of action of YiiP (Coudray *et al.*, 2013 ▸). In mechanistic descriptive terms, the cryo-EM structure of SoYiiP reveals a cytoplasmic/inward-facing state, whereas the X-ray crystal structure of EcYiiP in the presence of Zn^2+^ adopts a periplasmic/outward-facing conformation (Fig. 5 ▸ *a*). Comparison of these two structures led to a revised mechanism (Coudray *et al.*, 2013 ▸). In contrast to the scissoring model proposed by Lu and coworkers, this alternating-access mechanism proposed that both helical bundles (TM1–TM2–TM4–TM5 and TM3–TM6) swing around the Zn^2+^ ion, leading to changes in access to the Zn^2+^-binding site (Fig. 5 ▸ *b*). Regarding the CTD, it was also suggested that owing to very high affinity binding, four Zn^2+^ ions are likely to be present in binding site C (despite the lack of Zn^2+^ in the crystallization medium) and these may stabilize the dimeric structure of CTD, which remains static (Fig. 4 ▸ *b*).

Investigation of Zn^2+^ transport by YiiP using a proton gradient has been proposed (Gupta *et al.*, 2014 ▸). Using X-ray-mediated hydroxyl radical labelling and mass-spectrometry techniques to elucidate water accessibility within the TMD of the YiiP cavity, Gupta and coworkers found that Leu152, located in TM5, acts as a gate in the inter-cavity, leading them to propose a proton-coupled zinc-transport mechanism. According to this mechanism, opening the Leu152 gate in the inward-facing conformation exposes the substrate-binding site to the intracellular side, where Zn^2+^ is present. The binding of Zn^2+^ triggers the reorientation of TM5 and the closing of the Leu152 gate, which changes the conformation to the outward-facing state (Fig. 5 ▸ *c*). The proposed mechanism is coupled to protonation of His153, with a change in its proton­ation state dependent on the physiological pH gradient. Deprotonated His153 in a relatively alkaline cytosol tends to bind Zn^2+^, whereas a protonated His153 in a relatively acidic periplasm may facilitate Zn^2+^ release (Gupta *et al.*, 2014 ▸).

A parallel study by Shusterman and coworkers also demonstrated that human ZnT1 extrudes zinc across the plasma membrane by utilizing the electrochemical proton gradient. In their experiments, the rate of Zn^2+^ efflux decreased when the cytosolic pH became acidified and increased when the pH was alkalinized, indicating a pH-driven transport process. When their data were fitted to the Michaelis–Menten equation, the apparent *K*_m_ was pH 6.8 ± 0.2 (Shusterman *et al.*, 2014 ▸).

Although the above-mentioned studies started to elucidate the details of the molecular mechanism of action of zinc transporters, a recent study reporting the cryo-EM structure of SoYiiP at 4.1 Å resolution provided further insights (Lopez-Redondo *et al.*, 2018 ▸). Based on intermolecular cysteine cross-linking data, Lopez-Redondo and coworkers provided evidence that scissoring motions of the TMDs were not essential for Zn^2+^ transport. Rather, they propose that the splaying of the TMDs observed in the X-ray structure may be a consequence of either membrane-protein destabilization caused by detergent micelles or a difference in crystal contacts between the different crystal structures.

Another feature observed in the higher resolution SoYiiP structure was the presence of Zn^2+^ in binding sites A, B and C, similar to the X-ray structure. This observation suggests that both structures represent a bound state of YiiP and that the conformational changes reported for the alternating-access mechanism reflect an equilibrium between inward-facing and outward-facing conformations of a zinc-bound state (Lopez-Redondo *et al.*, 2018 ▸).

This new model proposes that alternating access to the Zn^2+^-binding site is established by rearrangement of the four-helix TM bundle against the TM3–TM6 bundle and the CTD, which acts as a scaffold. This type of alternating-access model can be described as a rocking-bundle mechanism, which has been observed in unrelated transporters such as LeuT (Lopez-Redondo *et al.*, 2018 ▸; Drew & Boudker, 2016 ▸; Fig. 4 ▸ *c*). To date, it is unclear how the two major conformations are connected by intermediate, occluded states.

Lopez-Redondo and coworkers also evaluated the role of the residues involved in the salt bridge and the binuclear Zn^2+^ site in the CTD, which have been described as essential for dimer stabilization. Mutants that disrupted the salt bridge and the Zn^2+^ binding between the CTDs all eluted as dimers in size-exclusion chromatography–multi-angle light-scattering (SEC-MALS) experiments, suggesting that other elements mediate YiiP dimerization. Analysis of the cryo-EM structures of SoYiiP showed that their TMDs have a larger contact area than in the EcYiiP crystal structure, and that this interface includes TM3 and the residues at the periplasmic end of TM1 and TM2. Taken together, these observations indicate that the TMD interface may also contribute to dimer stabilization (Lopez-Redondo *et al.*, 2018 ▸).

## 6. What is the role of the C-terminal domain?   

The CTD has been thought to play an important role in the mechanism of action of proteins belonging to the CDF family. The first reported crystal structure of the CTD of the CzrB protein from *T. thermophilus* was solved in the presence and absence of zinc, and both structures formed a ‘V-shaped’ homodimer (Cherezov *et al.*, 2008 ▸). Each protomer of the soluble CTD fragment of CzrB comprises three β-strands and two α-helices, resembling a metallochaperone fold (Figs. 2 ▸ *a* and 6 ▸). Importantly, and differing from the crystal structure of EcYiiP, four zinc ions were identified in the Zn^2+^-bound crystal structure of the CzrB CTD. Three of these are thought to be physiologically relevant, whereas the fourth is likely to be a crystallization artefact (Cherezov *et al.*, 2008 ▸).

**Figure 6:**
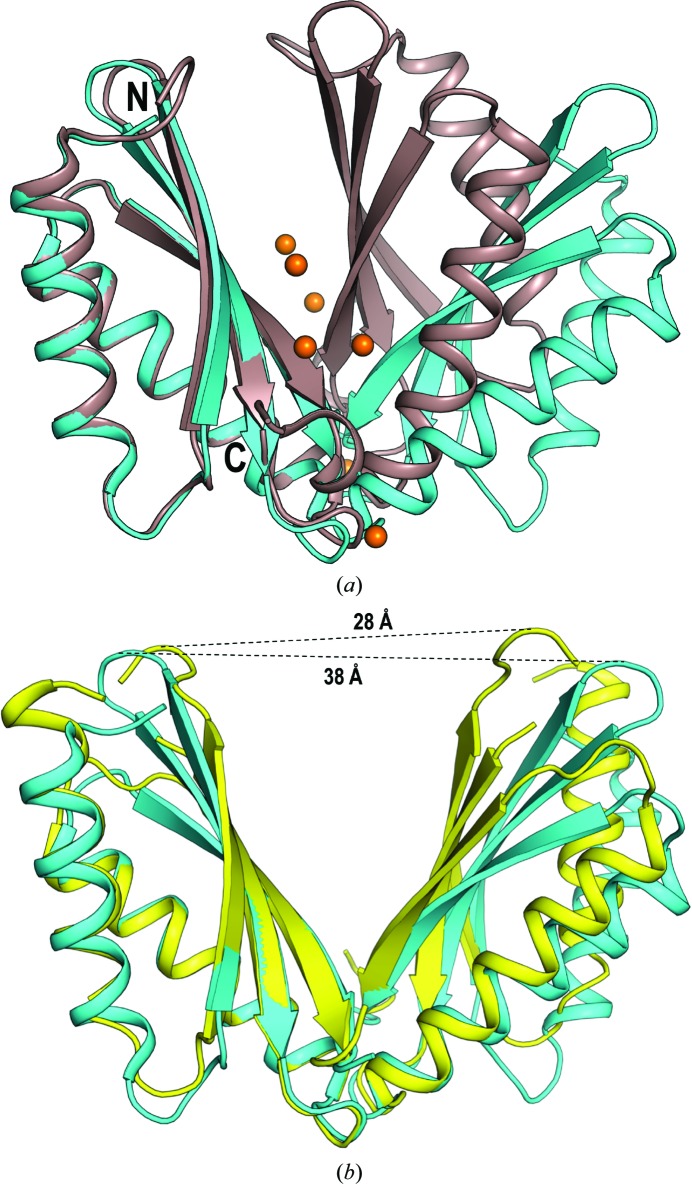
Comparison of CTD structures. (*a*) Structural comparison of the apo-form (cyan) and Zn^2+^-bound (brown) structures of the CTD of CzrB from *T. thermophilus* (PDB entries 3byp and 3byr; Cherezov *et al.*, 2008 ▸). Upon Zn^2+^ binding the CzrB CTD adopts a more compact and rigid structure. Zn^2+^ ions are shown in orange. The N- and C-termini are indicated. (*b*) Dimer structures of the apo CzrB CTD (cyan) and the apo TM0876 CTD (yellow; PDB entry 2zzt). Structural superposition suggests flexibility at the ‘top’ of the ‘V-shaped’ CTD in the absence of bound metal. The distance between equivalent residues in the two structures is indicated by a dashed line.

Comparison of apo and Zn^2+^-bound CzrB CTD structures suggests that the protein undergoes conformational changes upon metal binding, adopting a more compact dimeric structure (Cherezov *et al.*, 2008 ▸; Fig. 6 ▸ *a*). Higuchi and coworkers have reported that in the crystal structure of another CDF family member, TM0876-CTD from *Thermotoga maritima*, the CTD of the apo-form structure opens at a different angle when compared with the apo CzrB CTD structure (Higuchi *et al.*, 2009 ▸). The distance between the Arg244 residues in the protomers of the CzrB CTD dimer is 38 Å, whereas the corresponding distance (for Gly242) in TM0876 CTD is 28 Å (Fig. 6 ▸ *b*). This finding suggests flexibility in the CTD, at least in the absence of Zn^2+^, and this flexibility might affect the regulation of ion transport (Higuchi *et al.*, 2009 ▸). Further corroboration of this result was provided by novel studies on the CTD of MamM, a magnetosome-directed ion transporter belonging to the CDF family (Uebe *et al.*, 2011 ▸; Zeytuni *et al.*, 2014 ▸). The dimeric apo MamM CTD crystal structure at a resolution of 1.95 Å adopts a V-shape similar to those of CzrB and TM0876. Although the metal-bound structure of the MamM CTD could not be solved, small-angle X-ray scattering (SAXS) of the MamM CTD indicated a tighter and more compact arrangement upon metal binding (Zeytuni *et al.*, 2014 ▸), as also shown for the CzrB CTD. Moreover, molecular-dynamics simulation of the MamM CTD showed that the base of the V-shaped structure is rigid and stable, whereas the top of the V has increased flexibility, suggesting that several conformations could be accessed while ‘searching’ for the metal ion (Zeytuni *et al.*, 2014 ▸). Taken together, these data suggest that upon binding divalent metals the CTD undergoes a conformational change to a tighter and more static conformation that somehow facilitates ion transport through the TMD (Zeytuni *et al.*, 2014 ▸). In the light of a rocking-bundle mechanism it is plausible that Zn^2+^ binding rigidifies the CTD, which might then restrict the movement of TM3 and TM6. These two helices together with the CTD could act as a scaffold to aid conformational changes of TM1–TM2–TM4–TM5. Indeed, no major conformational changes were revealed in the structure of the MamB CTD after soaking with zinc (Uebe *et al.*, 2018 ▸), corroborating the SoYiiP structure. These findings would be consistent with a stable CTD, with conformational changes occurring solely in the TMD (Lopez-Redondo *et al.*, 2018 ▸). Taken together, these studies point out some simil­arities of CTDs, and suggest that the function of this domain might vary among bacterial homologues.

## 7. Concluding remarks   

The last five years have contributed enormously to a better understanding of the structure and function of the CDF family, particularly the zinc transporters. The recent structures of YiiP homologs (EcYiiP and SoYiiP), suggesting a transition between the inward-facing and outward-facing conformations of the zinc-bound form, reported different arrangements that shed light on possible mechanisms of action for this class of proteins. However, several questions need further investigation in order to fully understand the Zn^2+^/H^+^ antiporter mechanism.

(i) What are the structures of the intermediate-occluded and apo (inward-facing and outward-facing) states?
(ii) How are deprotonation and Zn^2+^ binding coupled?
(iii) How does Zn^2+^ binding induce conformational change?
(iv) Which residue(s) are involved in proton binding and release? His153 has been proposed, although experimental data are required to support this hypothesis.
(v) Which residues or regions are important for dimerization?
(vi) What is the function of the CTD?

Furthermore, we cannot rule out the possibility that the differences reported in the SoYiiP and EcYiiP structures could be attributed to (i) the use of distinct techniques to solve the structures (X-ray *versus* cryo-EM), (ii) distinct protein environ­ments (detergent micelles *versus* lipid bilayer) or (iii) distinct organisms: as mentioned above, the three-dimensional structures of the CTDs showed variations among the bacterial homologues.

Discrepancies have also been noted regarding protein motions within the CTD interface upon zinc binding. According to Lu and coworkers, in the presence of Zn^2+^ the EcYiiP CTD closes in a scissor-like fashion at the C terminus (Fig. 4 ▸ *a*). On the other hand, Cherezov and coworkers reported that in the presence of Zn^2+^ the soluble domains of CzrB (40% sequence similarity to the EcYiiP CTD) close at the N terminus (Fig. 6 ▸ *a*). More recently, Uebe and coworkers observed minimal changes in the structures of apo and Zn^2+^-bound MamB (Uebe *et al.*, 2018 ▸). These observations show that variations occur among this class of proteins as to how Zn^2+^ affects CTD structure and motion, and raise questions as to whether the movements within the CTD are organism dependent.

Metal-selectivity studies have shown that whilst some CDFs are very selective for a single metal, others can transport more than one metal. The studies in this review focused mainly on the role of the tetrahedral motif in binding site A. However, very little is known about the functional significance of binding sites B and C, and their role in metal specificity and selectivity. Moreover, the mechanisms of ion coordination and metal transport by ZnT and other CDF members remain unclear.

An overarching theme throughout many of these studies is that further structural, biophysical and functional elucidation of CDF members, from diverse organisms, and especially from eukaryotic organisms, is essential to furthering our knowledge into the mechanism of action of this family. Such information is essential to provide a better understanding of the biological processes that these proteins support and to provide templates for structure-based drug discovery targeting zinc-transporter disorders.
